# Affective and cognitive brain-networks are differently integrated in women and men while experiencing compassion

**DOI:** 10.3389/fpsyg.2022.992935

**Published:** 2022-09-13

**Authors:** Geraldine Rodríguez-Nieto, Roberto E. Mercadillo, Erick H. Pasaye, Fernando A. Barrios

**Affiliations:** ^1^Movement Control and Neuroplasticity Research Group, Department of Movement Sciences, Biomedical Sciences Group, KU Leuven, Leuven, Belgium; ^2^Unidad Iztapalapa, Universidad Autónoma Metropolitana, México City, Mexico; ^3^Consejo Nacional de Ciencia y Tecnología, México City, Mexico; ^4^Instituto de Neurobiología, Universidad Nacional Autónoma de México, Queretaro, Mexico

**Keywords:** compassion, empathy, moral, gender differences, functional connectivity

## Abstract

Different theoretical models have proposed cognitive and affective components in empathy and moral judgments encompassing compassion. Furthermore, gender differences in psychological and neural functions involving empathic and moral processing, as well as compassionate experiences, have been reported. However, the neurobiological function regarding affective and cognitive integration underlying compassion and gender-associated differences has not been investigated. In this study, we aimed to examine the interaction between cognitive and emotional components through functional connectivity analyzes and to explore gender differences for the recruitment and interaction of these components. Thirty-six healthy participants (21–56 years; 21 women) were exposed to social images in an fMRI session to judge whether the stimuli elicited compassion. The results showed a different connectivity pattern for women and men of the insular cortex, the dorsomedial prefrontal cortex (dmPFC), the orbitofrontal cortex (OFC), and the cingulate cortex. The integration of affective and cognitive components follows a complex functional connectivity pattern that is different for both genders. These differences may indicate that men largely make compassionate judgments based on contextual information, while women tend to notably take internal and introspective processes into account. Women and men can use different affective and cognitive routes that could converge in similar learning of moral values, empathic experiences and compassionate acts.

## Introduction

Compassion can be described as a feeling of affliction that is elicited by perceiving the pain or suffering of another and that motivates to alleviate the suffering party (Haidt, 2003). Since this moral emotion has been related to prosocial behaviors, such as altruism or caring, there has been a growing scientific interest in understanding its complexity from multiple perspectives. For example, its evolutionary origins, its neurobiological substrates and its relations with emotional and behavioral domains such as love, reconciliation, or cooperation (Goetz et al., 2010; Kim et al., 2020; Novak et al., 2022). Social and psychosocial perspectives have also contributed to understanding the phenomenology behind compassion and the sociocultural properties that influence variations in conceptual understanding and/or behavioral expressions (Keltner et al., 2010; Kariyawasam et al., 2021). As reasoned from the previous lines, compassion implies empathic abilities that allow inferring the suffering of others, as well as judgments, evaluation of social signals and decision making to perform helping behaviors. Thus, both affective and cognitive components shape and motivate compassionate experiences and actions.

Empathy and compassion must not be confused. Empathic inference about others states is not restricted to suffering but includes a variety of feelings, whether positive or negative. Also, compassion involves emotional and behavioral understandings, expressions and actions framed on necessary socio-cultural contexts (Preckel et al., 2018). So, empathy may be considered as a crucial affective component of compassion. This component may be phylogenetically recent and emerge early during human development (Perner, 1992; Diamond, 2002). Its neurobiological substrates involve a sensorimotor mirror system particularly based on the anterior insula (AI) and on other brain regions such as, the anterior cingulate cortex (ACC) and the inferior frontal gyrus (IFG) engaged during the first-hand experience of pain and disgust and when perceiving someone else experiencing similar physical or emotional states (Singer et al., 2006; Jabby and Keysers, 2008; Van Overwalle, 2009; Zaki et al., 2009; Lamm et al., 2011).

The cognitive components that shape compassion may have emerged later in evolution and allow humans to better understand and speculate about the intentions and internal states of others. Brain regions with social perception and mentalizing-related functions are proposed as part of such components: the superior temporal sulcus, the medial prefrontal cortex, and the temporoparietal junction (Lamm et al., 2007; Decety and Svetlova, 2012; Healey and Grossman, 2018). In particular, the dorsomedial prefrontal cortex (dmPFC) has been proposed as the critical region involving a network for mentalizing and high-level constructional processes for social stimuli, social learning, and decision-making that allow complex social behaviors (Baetens et al., 2017; Alcalá-López et al., 2018; Moll et al., 2018; de Kloet et al., 2021; Ni and Li, 2021).

The orbitofrontal cortex (OFC) and the anterior cingulate cortex (ACC) have been proposed to play a critical role in affective-cognitive integration (Decety and Svetlova, 2012). OFC damage leads to antisocial behaviors and lack of empathy (Bechara et al., 2000; Damasio, 2003; Decety et al., 2012). As for ACC, its function is related to decision making and convergent information integration (Allman and Atiyahakeem, 2001; Botvinck et al., 2004; Yu et al., 2011).

The affective and cognitive components involving compassion are not the only interesting issue. Gender differences remain a controversial field. For example, women tend to express empathic concern (Reyes-Aguilar and Barrios, 2016) and care-oriented decisions to a greater extent when they reason a sense of injustice, while men tend to express duty-oriented thoughts when reasoning morally (Björklund, 2003; Edele et al., 2013). Likewise, activation in the posterior cingulate cortex and the AI occurs in women while the inferior parietal cortex occurs in men in response to moral stimuli; these activations are related to the perceived severity of a moral violation (Harenski and Hamann, 2006; Harenski et al., 2008). Controversially, although women score higher than men on self-reported dispositional empathy when viewing scenes of induced physical pain, no gender differences are found in brain-related activation of empathy involving the amygdala, the prefrontal cortex, the IA and the ACC (Michalska et al., 2013). Regarding compassion, two studies report that women and men express similar compassionate experiences while viewing compassion-evoking images, but women show greater and more diverse activation than men in the ACC, the left superior frontal gyrus, the thalamus, the insular cortex, and the prefrontal cortex (Mercadillo et al., 2011, 2015a).

Research on the neural basis of compassion using neuroimaging has included a variety of designs. For example, listening to stories and imaginary about situations of suffering (Kédia et al., 2008; Immordino-Yang et al., 2009), or reading statements and observing visual stimuli (Moll et al., 2003; Kim et al., 2009). Such experimental diversity shows a consequent variety of neurobiological findings whose cognitive and affective functions we are still discussing. By a meta-analysis derived from 16 fMRI studies on compassion, Kim et al. (2020) showed common activation in the inferior frontal gyri, the substantia nigra/periaqueductal gray, the ACC, the AI, the putamen, and the thalamus when experiencing compassion elicited by different sensory modalities. Novak et al. (2022) presented a systematic review of 35 neuroimaging studies revealing that the IFG, the cerebellum, the middle temporal gyrus, the insula, and the caudate nucleus are the most recurrent brain regions associated with compassion. Although these reports indicate neuroscientific interest in compassion, analyzes focused on anatomical location and/or brain activation elicited when performing tasks, but functional connectivity and gender differences have not been assessed. It remains unclear whether affective and cognitive components integrating compassion are anatomically and functionally dissociable or may be independent and recruit overlapping brain functions. Furthermore, it is imprecise whether compassion brain-related functions are similarly or distinctively recruited by women and men, even if similar compassionate experiences are expressed.

We present an exploratory study assessing gender differences when watching compassion-evoking pictures and indicating compassionate experiences motivating helping behaviors. Our approach is based on functional brain connectivity using Psychophysiological interaction (PPI) analysis (O’Reilly et al., 2012) focused on four brain regions: Right AI as a crucial affective component for compassion due to its recurrent activation when perceiving suffering inflicted on others (Singer et al., 2006; Lamm et al., 2011); right-dmPFC as a cognitive component due to its role in high-level processes and mentalization required for social learning and decision-making that favor compassionate expressions (Baetens et al., 2017; Ni and Li, 2021); left-ACC and OFC as brain integrators due to their proposed role in the convergence of both affective and cognitive information involving social situations (Allman and Atiyahakeem, 2001; Decety and Svetlova, 2012). These four brain regions were reported to be functionally active in a previous study using the same experimental task as the one used here (Mercadillo et al., 2011).

## Method

### Participants

Thirty-six participants (21 women, M age = 34 ± 9.9, range: 21–56 years; 15 men, M age = 31 ± 9.2 years, range: 20–52 years) were recruited through advertisements in internet groups and through personal invitations in Mexico City and Querétaro (Mexico). Since most studies on the functional brain basis of compassion are limited to college-educated youth, we aimed to recruit a more diverse sample for this exploration. An inclusion criterion was 12 years of education, which in Mexico is considered basic education (9 years) and high school (3 years) to promote adequate reading ability, as well as understanding of instructions and information about the experiment. Criteria also included strong right-handedness as measured by the Edinburgh Handedness Inventory, good general health as verified by a clinical interview, and the absence of current mental and neurological disorders as assessed by the Mexican electronic version of the Symptom Check List 90 (González-Santos et al., 2007) and a psychiatric interview. Security restrictions for magnetic resonance imaging studies were also considered. The protocol was designed in accordance with the guidelines of the American Psychological Association (2002) and the Declaration of Helsinki and was approved by the Bioethics Committee of the Institute of Neurobiology of the Universidad Nacional Autónoma de México. No individual was paid for their participation. No subject was taking any regular medication during any stage of the study.

### Experimental task

The task was designed in E-Prime (Psychology Software Tools, Inc., Pittsburg, PA, United States) and projected through the visual system with googles placed on the head coil (Nordic Neurolab, Bergen, Norway). It consisted of one series of 100 visual stimuli from the International Affective Picture System (Lang et al., 2005) previously validated by our group for fMRI studies on compassion in Mexican samples (see Mercadillo et al., 2007, 2011, 2015a).

**FIGURE 1:**
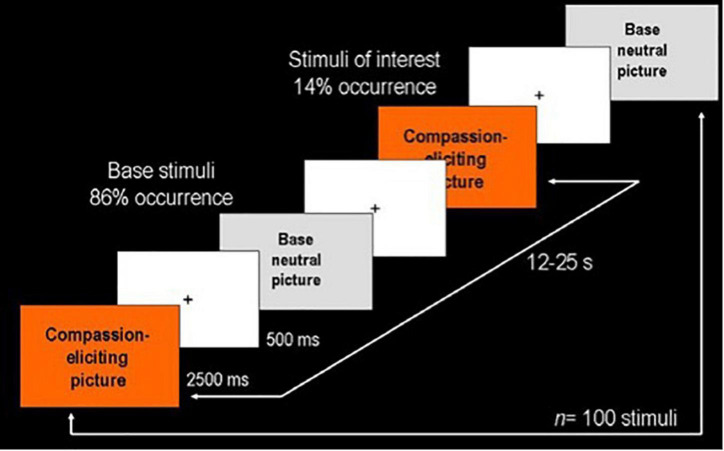
Event-related design used in the presentation of visual stimuli. The series consisted of 100 pictures: 14 compassion-eliciting pictures depicting suffering in different contexts (stimuli of interest) and 86 neutral pictures representing common social scenes (base stimuli). Each stimulus was presented for 2,500 ms followed by a fixation cross for 500 ms. Stimuli of interest were randomly presented at 12–25 s intervals.

Two categories of stimuli in the series were contrasted applying an event-related design. Fourteen compassion-evoking pictures depicting suffering in different settings and situations (e.g., war scenes, sad facial expressions, famine situations, or people experiencing poverty or addiction) were alternated with 86 emotionally neutral social pictures (e.g., people walking or waiting for the bus). Each picture was presented for 2,500 ms followed by a fixation cross with 500 ms duration (Figure 1).

Participants were instructed to respond *via* a button box (ResponseGrip, Nordic Neurolab, Bergen, Norway) if each image elicited compassion (Response: Yes/No). Behavioral responses were recorded to verify attention during the task and to quantify stimuli reported as eliciting compassion. Compassion was defined as feelings of affliction caused by the perception of suffering in others that motivates helping the suffering party. To neutralize the effect of lateralized finger motor responses, half of the participants used their right index finger while the rest used their left.

### Imaging acquisition and data analysis

Participants were scanned in a GE Discovery MR750 3T scanner (General Electric Medical Systems, Milwaukee, WI, United States) at the Resonance Magnetic Unit, Institute of Neurobiology, Universidad Nacional Autoìnoma de Meìxico. Anatomical images were collected with a high-resolution 3D SPGR (spoiled gradient sequence); 140 slices, relaxation time = 24 ms, echo time = 5 ms, flip angle = 30°, voxel size = 1 × 1 × 1 mm^3^. Functional images were acquired using an EPI-GRE sequence (30 slices, 5 mm thick with no gap, relaxation time = 3000 ms, echo time = 30 ms, flip angle = 90°, FOV = 24 cm, voxel size = 4 × 4 × 4 mm^3^).

All preprocessing and statistical analyses were conducted using FSL 4.1.^1^ At the individual level, the first four data points of the run were discarded. Preprocessing of images included: time slice correction to synchronize for inter-slice time difference; MCFLIRT realignment for head movement (Jenkinson et al., 2002); spatial smoothing with a 6 mm FWHM Gaussian kernel (Friston, 2007); BET extraction (Smith, 2002); and normalization to the standard Montreal Neurological Institute (MNI) space.

Connectivity analyses were performed following a PPI method according to the procedures described in O’Reilly et al. (2012). Regions of interest (ROI) were defined on the basis of the activated regions mean map. An isometric mask (3 voxels^3^) at one of two possible locations (as some locations were not suitable for each individual participants) was located in each ROI for each participant: right AI (x, y, z = 42, 16, 0; 42, 22, −2), left ACC (x, y, z = −2, 16, 32; −2, 14, 34), left OFC (x, y, z = −44, 22, −4; −38, 28, −4), and right dmPFC (x, y, z = 4, 18, 48; 0, 18, 50).

Some participants with specific poor ROI structural co-localization from the MNI standard, with more than one voxel shift in the seed localization, were not used for that structure PPI functional connectivity estimation. The final sample for the functional connectivity analyses was: (r)AI: women = 17, men = 13; (l) ACC: women = 18, men = 14; (l) OFC: women = 16, men = 10; and (r) dmPFC: women = 19, men = 16.

A general linear model was used to analyze the interaction of the time course in each ROI and the presentation time points of compassion-evoking stimuli (PPI analysis) in the whole brain. Since PPI analyses tend to lack statistical power, especially in event related paradigms (O’Reilly et al., 2012), we decided to consider those results with a *P* < 0.005 threshold level and clusters shaped by seven contiguous voxels as minimum.

## Results

As indicated by the finger-motor responses, we did not observe gender differences regarding compassionate experiences elicited by watching the pictures (*n* = 14; men = 13 ± 1.37; women = 12.8 ± 1.08; *T* = 0.48, *p* = 0.92).

**FIGURE 2:**
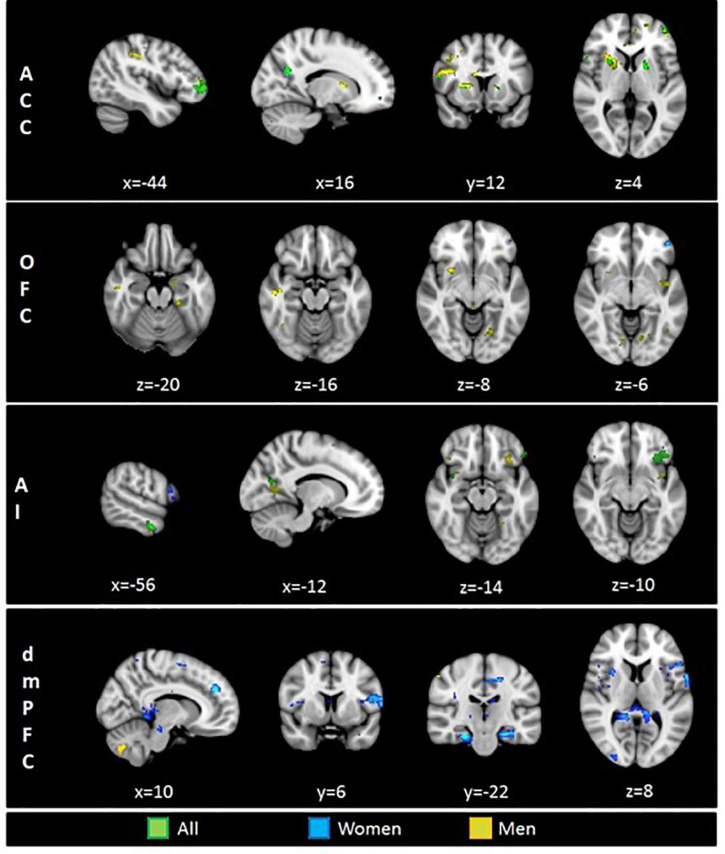
Brain regions functionally connected with four seed regions of interest (ROI): ACC, anterior cingulate cortex; OFC, orbitofrontal cortex; AI, anterior insula; dmPFC, dorsomedial prefrontal cortex. Displayed colors represent tree different groups: Green–Full sample, Blue–Women, Yellow–Men. Results at *p* < 0.005.

Psychophysiological interaction analyses for the ACC seed revealed a significant effect for the full sample integrating women and men in the frontal pole, the IFG, the precuneus, the putamen, and the lateral occipital cortex. The analysis for men showed a profuse connectivity with frontal and temporal regions. In addition, neural coupling with the post-central gyrus, the insular cortex, the central operculum, the putamen and the cerebellum were found. In women, the ACC seed showed to be functionally connected with the precuneus (Table 1 and Figure 2).

**TABLE 1:** Brain regions presenting significant functional connectivity with four different seed regions: anterior cingulate cortex (ACC), orbitofrontal cortex (OFC), dorsomedial prefrontal cortex (dmPFC), and anterior insula (AI).

	MNI coordinates				
Brain region	Laterality	*x*	*y*	*z*	*Z-*value
**Anterior cingulate cortex (seed)**					
*Full sample*					
Frontal pole	L/R	−46	48	−2	3.4
		14	54	−8	3.03
		46	38	−8	2.8
Precuneus	R	16	−60	26	3.3
Inferior frontal gyrus	L/R	−52	36	8	3.3
		54	16	4	3.16
Putamen	L/R	−18	6	4	3.2
		26	14	4	3.1
Lateral occipital cortex	R	30	−48	−38	2.8
Orbital cortex	R	42	26	−16	2.7
*Women*					
Precuneus	R	16	−62	28	2.5
*Men*					
Temporal pole	R	46	22	−30	3.3
Putamen	R	26	14	4	3.3
Inferior frontal gyrus	R	52	14	22	3.3
Post-central gyrus	R	50	−24	44	3.2
Cerebellum	R	32	−44	−42	3.2
Supramarginal gyrus–TPJ	L	−42	−32	40	3.1
		64	−20	24	3.1
Precentral gyrus	R	56	10	18	3.1
		26	−6	46	2.7
Middle temporal gyrus	L	−48	−4	−30	3.09
Insular cortex	R	38	0	10	3.08
Middle frontal gyrus	R	32	12	40	3.03
Frontal pole	L/R	−22	56	4	2.9
		50	40	10	2.9
		−6	56	−4	2.8
Inferior temporal gyrus	L	−48	−52	−18	2.8
Central operculum	L	−50	−22	−18	2.8
Superior frontal gyrus	R	24	14	50	2.7
Paracingulate cortex		0	54	4	2.7
**Orbitofrontal cortex (seed)**					
*Full sample*					
Null					
*Women*					
Frontal pole	L	−46	46	−6	2.8
*Men*					
Middle temporal gyrus	R	52	−14	−16	3.009
Putamen	R	28	10	−8	2.84
Amygdala	L	−20	−6	−22	2.7
Parahippocampal gyrus	L	−24	−32	−20	2.6
Fusiform gyrus	L	−22	−6	−8	2.6
**Anterior insula (seed)**					
*Full sample*					
Middle temporal gyrus	L	−56	−6	−28	3.7
Lingual gyrus–TPJ	L	−24	−54	−8	3.1
Precuneus	L	−10	−66	16	3.08
Cerebellum	L/R	−24	−48	−52	3.0
		46	−56	−34	3.0
Orbitofrontal cortex	L	−52	32	−14	2.9
Parahippocampal gyrus	L	−26	−28	−20	2.9
Fusiform cortex	R	30	−38	−22	2.9
Occipital lateral cortex	R	40	−66	12	2.8
		26	−80	24	2.8
Inferior frontal gyrus	R	52	22	4	2.7
*Women*					
Frontal pole	R	14	44	40	2.8
Inferior frontal gyrus	L/R	−56	18	10	2.5
		60	14	2	2.5
Middle temporal gyrus	L	−48	−8	−26	2.5
*Men*					
Orbitofrontal cortex	L	−34	24	−14	2.9
Anterior insula	L	−38	6	−12	2.8
Inferior temporal gyrus	L	−58	−8	−32	2.8
Precuneus	L	−6	−80	44	2.8
Intracalcarine fissure	L	−12	−62	6	2.8
Cerebellum	R	44	−52	−46	2.8
Temporal pole	L	−52	6	−32	2.7
Post-central gyrus	R	38	−20	40	2.7
Fusiform cortex	L	−24	−52	−18	2.7
Lateral occipital cortex	R	28	−80	24	2.7
Superior parietal lobe	R	26	−50	52	2.7
**Dorsomedial prefrontal cortex (seed)**					
*Full sample*					
Null					
*Women*					
Parahippocampal gyrus	R/L	24	−20	−24	3.7
		−30	−28	−18	3.26
Inferior frontal gyrus	L	−52	10	14	3.46
Precentral gyrus	L	−62	2	12	3.46
Paracingulate gyrus	R	10	44	30	3.43
Cerebellum	R/L	26	−54	−54	3.4
		−2	−46	−8	3.06
Posterior cingulate cortex	L	−6	−20	44	3.18
Amygdala	R	30	−6	22	3.05
Hippocampus	R/L	24	−26	−10	3.04
		−32	−18	−20	2.9
Insula	R	30	12	8	3.03
Lingual gyrus	R	12	−42	−4	3.01
Middle temporal gyrus	R	58	−2	−24	2.9
Central operculum	L	−40	8	12	2.8
Putamen	L	−24	−4	16	2.7
Thalamus	R/L	6	−32	4	2.7
		−8	−32	10	2.6
Precuneus	R	30	−50	66	2.5
*Men*					
Cerebellum	L/R	−26	−66	−48	3.04
		14	−74	48	2.87

Results given for *Full sample* integrating women and men, for only *Women* and for only *Men*.

When analyzing the functional connectivity for the OFC seed no significant effects were found for the full sample. For women, the OFC showed connectivity with the frontal pole. For men, this region showed functional connectivity with the middle temporal gyrus, the putamen, the parahippocampal and fusiform gyri, and the amygdala (Table 1 and Figure 2).

Functional connectivity analyses for the AI in the full sample revealed a profuse connectivity with a network involving the OFC, the IFG, the middle temporal gyrus, the fusiform gyri, the precuneus, the lateral occipital cortex and the cerebellum. For men, the AI showed to be connected with the OFC, the temporal pole, the post-central gyrus, the precuneus, the fusiform cortex, the lateral occipital cortex and the superior parietal lobe. Conversely, analyses for women revealed connectivity with the frontal pole, the IFG and the middle temporal gyrus (Table 1 and Figure 2).

In regard to the functional connectivity for the dmPFC seed, no significant effects were observed for the full sample. For women, the dmPFC displayed a wide-spread connectivity with cortical and subcortical regions including the IFG, the middle temporal gyrus, the insular cortex, the central operculum, the parahippocampal gyrus, the posterior cingulate cortex, the precuneus, the cerebellum, the putamen, the hippocampus, and the amygdala. For men, the dmPFC presented a neural coupling with different cerebellar clusters (Table 1 and Figure 2).

## Discussion

In this exploratory study, we aimed to assess gender differences in the affective and cognitive components underlying compassion. We examined the functional connectivity presented in four brain regions related to those components (AI and dmPFC, respectively), as well as to affective-cognitive integration (ACC and OFC). We did not find a solid pattern of connectivity that supports the role of the ACC or the OFC as the main affective-cognitive integrators. However, despite the extensive overlap in brain activation reported for women and men while experiencing compassion (Mercadillo et al., 2011, 2015a), we clearly found dissociable connectivity patterns for both genders suggesting distinctive neurocognitive pathways that allow compassionate experiences and decisions.

We expected that the OFC and/or the ACC could play as integrators of affective and cognitive components. Our results may support this assumption only for the ACC in men for whom it was connected with the IA, the IFG (affective component-associated brain regions), the dmPFC (related to mentalizing), and with other regions related to social and moral cognition, such as the frontal and temporal poles. As previously suggested, that ACC connectivity may allow regulation of empathic expressions (Kunz et al., 2011; Olalde-Mathieu et al., 2022) and its connectivity with the temporal pole may implicate autobiographical processes and the attribution of social qualities in others (Mercadillo et al., 2017). For women, the ACC was only coupled to the precuneus, whose function involves self-awareness related to emotional valuations, episodic memory (Ochsner et al., 2004; Atilano-Barbosa et al., 2022), imagery about another’s mental states (Schurz et al., 2014) and moral judgments (Bzdok et al., 2012). The precuneus is also suggested as a central node in fronto-parietal networks allowing connectivity between different brain regions (Bullmore and Sporns, 2009). Thus, the precuneus may function within a cascade-like mechanism that gathers information from other brain functions and leads to compassionate integration with salient introspective processes in women.

The OFC seed did not exhibit connectivity with neither brain regions involving affective nor cognitive components for the entire sample. However, for men, the coupling with the amygdala, the parahippocampal cortex, the putamen, and the middle temporal gyrus may suggest a role for the OFC as an integrator of emotional and mnemonic elements, as suggested when people feel anger or sadness while making moral judgments on collective painful situations (Fourie et al., 2017). In women, the OFC exhibited functional connectivity only with the frontal pole, which was also connected to the IA in women and to the ACC in the full sample. The frontal pole may play an important role in moral cognition, values, and long-term goals; furthermore, it exhibits structural and functional differences between long-term loving-kindness meditation practitioners (Greene and Haidt, 2002; Moll et al., 2005; Moll and Schulkin, 2009; Engen et al., 2018). The observed patterns of connectivity may suggest the integration of long-term values that encompass the affective component of compassion, as well as, moral appraisals while experiencing compassion involving beliefs and learned values.

Regarding the AI as an affective component, its connectivity with the IFG for the full sample and for women may imply a mirror system that allows mimicry of gestures and emotional contagion (Jabby and Keysers, 2008). The profuse connectivity between the AI and occipital regions, both for the full sample and for men, may suggest visual input influencing somatovisceral responses, presumably related to pain. Only men showed functional connectivity between the AI and the temporal pole, with functions proposed for the understanding of social semantics (Moll et al., 2005) and for the integration of higher order information that involves emotional-visceral responses (Olson et al., 2007). For the full sample, the IA presented connectivity with the middle temporal gyrus. Interestingly, the ACC and OFC were also connected to this region for men, while for women it was connected to the dmPFC. Damage in the middle temporal gyrus has been associated with decreased altruistic behaviors in an experiment on real charitable decisions (Moll et al., 2018). Further studies may investigate whether the strength of the connectivity patterns of the middle temporal gyrus can predict altruistic decision making.

In contrast with the profuse connectivity revealed for the ACC and the AI as seed regions for men, women showed a more spread connectivity from the dmPFC. The dmPFC connectivity with the IFG and the central operculum is remarkable since their role in mimicry and emotional contagion suggest a mirror system directly intervening in the inference of other’s mental states. In addition, the dmPFC showed connections with the parahippocampal gyrus and posterior cingulate with functions related to scene recognition and emotional salience in episodic memory (Epstein and Kanwisher, 1998; Maddock et al., 2003). This connectivity pattern may exchange information among different cognitive sources required for mentalizing.

It is suggested that the dmPFC together with the posterior cingulate cortex modulated severity values in moral judgments; modulation by the posterior cingulate cortex has been reported to be significantly stronger in women than in men (Harenski and Hamann, 2006; Harenski et al., 2008). Robertson et al. (2007) report greater posterior cingulate activation when making care-based judgments compared to fairness-based judgments. So, women may perform compassionate judgments in a more caring-based way that requires inner elements, such as self-reflection or episodic memory. A notable finding is the dmPFC connectivity with the hippocampus and the amygdala for women. This connectivity may suggest a role for the dmPFC as a Theory of Mind or mentalization node assembling mnemonic and emotional information required for social decisions, such as expressions of distress and aversive situations in social contexts presented in the design of the task (Mercadillo et al., 2015a).

Both women and men showed connectivity between the dmPFC and the cerebellum, although more extensively for men. In recent years, neuroimaging findings have highlighted the role of the cerebellum in affective processes and experiences (Baumann and Mattingley, 2012). Furthermore, the reciprocal connections of the cerebellum with the prefrontal and cingulate cortices point to its relevance for moral cognition (Demirtas-Tatlidede and Schmahmann, 2013). Clinical approaches have reported that cerebellar damage causes alterations in mentalization, empathy, and social cognition (Gerschcovich et al., 2011; Mercadillo et al., 2015b). The relevance of the cerebellum for compassion may depend on sensory input, and how much it affects higher-order cognition remains unknown. We suggest that the cerebellum modulates unconscious bodily behaviors relevant to social or interpersonal dynamics and, in turn, is modulated by information or emotional experiences. Modulated behaviors may include gaze direction, posture, and language needed to infer states of distress in others and express compassion when caring or helping. Further connectivity analyzes with cerebellar seed regions would be helpful in exploring new neurobiological approaches to compassion.

The profuse functional connectivity of the ACC and the IA for the full sample may suggest common neural processes denoting affective components and the integration of affective-cognitive elements for compassion based on said brain regions. This cannot be said for the OFC and the dmPFC connectivity; null effects for the full sample suggest that the patterns identified separately for each gender are so different that they vanished when analyzed together.

**FIGURE 3:**
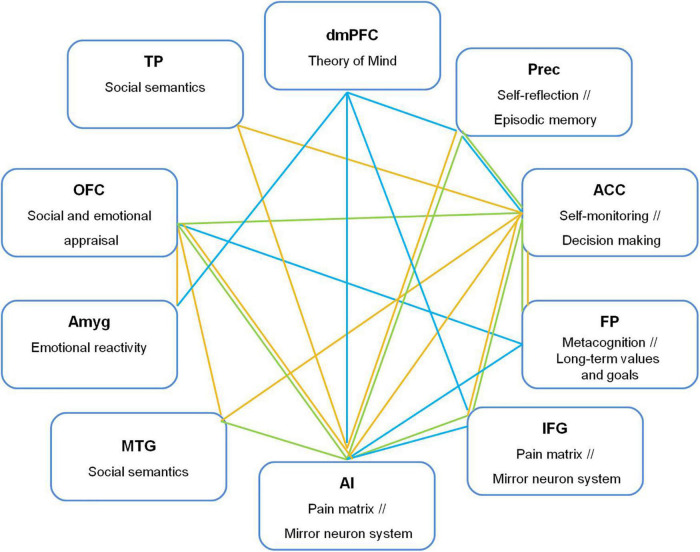
Functional connections from dorsomedial prefrontal cortex (dmPFC), anterior cingulate cortex (ACC), orbitofrontal cortex (OFC), and anterior insula (AI) with brain regions related to empathic and moral processes underlying compassion. Prec, precuneus; FP, frontal pole; IFG, inferior frontal gyrus; TPJ, temporoparietal junction, Amyg, amygdala; OFC, orbitofrontal cortex; TP, temporal pole; MTG, middle temporal gyrus. Green line: Both-gender group; yellow line: Men, blue line: Women.

The differences in functional connectivity found for women and men suggest a more complex system than the expected affective-cognitive integration based on one or two brain regions, such as the OFC or the ACC. The interpretation of these gender differences must consider several anatomical and behavioral elements. Women have been reported to exhibit greater anatomical connectivity, resulting in more diversified pathways that can make pattern identification more difficult (Gong et al., 2009). Regarding behavior, previous findings show that women’s empathy and moral judgments involving compassion predominantly recruit introspective and affective resources, whereas men may primarily use attentional processes and contextual information to define social cues involving compassion (Björklund, 2003; Singer et al., 2006; Harenski et al., 2008; Mercadillo et al., 2011; Moriguchi et al., 2014). It is possible, then, that a more profuse connectivity of the IA and the ACC for men underlies a modulating role of contextual factors in affective response and deliberation of possible helping behaviors. Importantly, these gender differences do not necessarily imply different consciously communicated compassionate experiences or prosocial motivated or performed behaviors when experiencing compassion. The differences may imply that women and men use different affective and cognitive routes that could converge in similar learning of moral values, empathic experiences and compassionate acts. How human evolution has determined such differences and how they depend on a particular gender-differentiated education or social context influencing functional connectivity requires further analytical research that can extend the neuroimaging findings. For now, we provide a summary of our findings in Figure 3 to be useful in further studies on affective and cognitive hypotheses about compassion based on functional connectivity.

Our study has several limitations. We cautiously expect that the effects reported here are strong enough to be significant despite the small sample size. However, large samples are needed in functional connectivity studies to reduce the effect of individual variability that can lead to false positives. Therefore, a larger sample is necessary to confirm our results. Our sample included a wide age range and flexible selection criteria with the intention of exploring the neurobiology of compassion not limited to young and highly educated populations. However, certain conditions may have caused unknown effects. For example, controversial age-related differences in empathy have been reported (Lamm et al., 2018; Wieck et al., 2021; Ziaei et al., 2021). Additionally, a progressive decline in functional connectivity has been reported for default mode, ventral attention, and sensorimotor networks, while increased connectivity in the visual network for individuals older than 50 years (Zonneveld et al., 2019). Therefore, larger samples considering age groups can be used for comparisons in future studies. Likewise, further research could be done considering behavioral assessments, such as empathic dispositions, cooperative attitudes, moral reasoning, or educational level to relate them to functional connectivity patterns.

## Data availability statement

The raw data supporting the conclusions of this article will be made available by the authors, without undue reservation.

## Ethics statement

The studies involving human participants were reviewed and approved by Bioethical Committee of the Institute of Neurobiology, Universidad Nacional Autoìnoma de México. The patients/participants provided their written informed consent to participate in this study.

## Author contributions

GR-N, RM, and FB developed the study concept and data analysis and interpretation. GR-N and EP performed testing and data collection. GR-N and RM drafted the manuscript under the supervision of FB. All authors contributed to the study design and approved the final version of the manuscript for submission.

## Footnotes

1
www.fmrib.ox.ac.uk/fsl
